# Dual-Strain Psychobiotics Combining Live *Lactiplantibacillus plantarum* PS128 and Heat-Treated *Lacticaseibacillus paracasei* PS23 Improve Psychological and Neuroendocrine Outcomes in Stressed Adults: A Randomized, Placebo-Controlled Trial

**DOI:** 10.3390/foods14244190

**Published:** 2025-12-06

**Authors:** Mon-Chien Lee, Ting-An Lin, Chi-Chang Huang

**Affiliations:** 1 Graduate Institute of Sports Science, National Taiwan Sport University, Taoyuan City 333325, Taiwan; 2 Center for General Education, Taipei Medical University, Taipei 11031, Taiwan

**Keywords:** probiotic, postbiotic, psychobiotics, stress, Neuralli Mood, overwork

## Abstract

Chronic psychological stress impairs neuroendocrine balance and increases the risk of mental health disturbances, including anxiety, sleep disruption, and low mood. The gut–brain axis has emerged as a promising target for stress modulation, particularly through psychobiotic interventions. This randomized, double-blind, placebo-controlled trial evaluated the effects of a combined psychobiotic formulation (Neuralli Mood), comprising live *Lactiplantibacillus plantarum* PS128 (PS128) and heat-treated *Lacticaseibacillus paracasei* PS23 (HT-PS23), on the psychological and physiological stress responses in a high-stress occupational population. A total of 116 healthy participants with elevated perceived stress (PSS ≥ 14), primarily firefighters, were randomly assigned to receive the dual-strain supplement or placebo for 8 weeks. Stress-related outcomes were assessed by using validated psychological scales and serum biomarkers. Compared with placebo, the psychobiotics group showed significantly greater reductions in overall job stress perception (JSS), state anxiety (STAI), and insomnia severity (ISI) (all *p* < 0.05). Additionally, serum adrenocorticotropic hormone (ACTH) and norepinephrine levels were significantly reduced post-intervention, whereas cortisol levels remained unchanged. These findings suggested that combining live and heat-treated psychobiotic strains may provide a safe and effective strategy for alleviating psychological stress and regulating neuroendocrine function in high-risk populations.

## 1. Introduction

Stress has become a pervasive psychophysiological challenge in contemporary society. It is defined as the integrated psychological and physiological response of an individual to perceived threats, often accompanied by anxiety, anticipation, and emotional fluctuations, which can significantly influence cognitive functions, such as learning and memory [1]. As individuals spend a significant portion of their lives immersed in occupational activities, prolonged exposure to stressors such as overwork, high workloads, time pressure, organizational instability, and lack of autonomy has a profound impact on psychological well-being [2]. Although acute stress responses serve adaptive purposes, chronic stress disrupts neuroendocrine regulation and triggers maladaptive physiological changes. Central to these effects is the dysregulation of the hypothalamic–pituitary–adrenal (HPA) axis, which governs the release of glucocorticoids and orchestrates systemic stress responses [3]. Sustained HPA axis activation compromises the balance of the autonomic, immune, and metabolic systems, contributing to impaired neuroplasticity, elevated neuroinflammation, and altered affective processing [4]. These physiological perturbations not only underlie the onset and persistence of anxiety, depression, and burnout syndromes but also highlight the need to identify integrative biological pathways that bridge environmental stress and mental health deterioration [1].

Among the biological systems implicated in stress regulation, the gut microbiota has garnered increasing attention as a potential modulator of host neurophysiology [5]. Through its role in the gut–brain axis, a complex bidirectional network involving neural, endocrine, and immune communication, the intestinal microbiota is believed to influence behavioral and emotional outcomes under stress [6]. To avoid redundancy, we provide here only the essential GBA context most relevant to the current study. Microbial metabolites and structural components may interact with host signaling systems to modulate HPA axis reactivity, immune tone, and intestinal barrier integrity [7]. These pathways are thought to affect the synthesis and availability of neuroactive compounds, such as serotonin, dopamine, and gamma-aminobutyric acid (GABA), many of which originate from the microbial metabolism of tryptophan and other dietary substrates [8]. Additionally, microbe-derived metabolites such as short-chain fatty acids (SCFAs) and secondary bile acids have been suggested to influence neuroinflammatory responses and possibly support neurotrophic signaling [9]. Notably, compositional differences in the gut microbiota have been observed between healthy individuals and those with mood-related disorders, suggesting a plausible therapeutic role for gut-targeted interventions [10].

Probiotics are live microorganisms that provide health benefits to a host [11]. Beyond their well-established roles in gastrointestinal health, such as maintaining microbial balance, reinforcing epithelial barrier function, and modulating immune responses, probiotics have gained attention for their neuroactive potential through the gut–brain axis [12]. This has led to the emergence of psychobiotics, a class of probiotics that influence mental health by modulating neurochemical, endocrine, and immune pathways related to stress regulation [13]. A subset of bacterial strains collectively termed psychobiotics has shown the potential to modulate HPA axis activity, immune parameters, and neurochemical dynamics, thereby alleviating stress-related symptoms [14]. Although these mechanisms remain unclear, the gastrointestinal tract is increasingly being viewed as a promising interface for psychological modulation. Animal studies have suggested that psychobiotic administration may preserve gut barrier function and counteract stress-induced microbial and immune alterations [15]. In human trials, 30 days of supplementation with *Lactobacillus helveticus* and *Bifidobacterium longum* has been associated with reduced psychological distress [16], whereas *Lactiplantibacillus plantarum* P8 has been shown to improve stress, anxiety, and sleep quality in individuals with a moderate psychological burden [17]. In addition, *Bifidobacterium breve* has demonstrated benefits in terms of emotional reactivity and cognitive function [18]. Despite this growing body of evidence, the psychological effects of probiotics remain highly strain-specific, and further investigation is required to characterize the functional properties and therapeutic applications of individual psychobiotic candidates.

*L. plantarum* PS128 (PS128) has been extensively studied and shown to exert beneficial effects on mood regulation, including significant reductions in anxiety, depression, and sleep disturbances [19,20,21]. Moreover, in high-stress occupational groups, such as information technology professionals, PS128 supplementation may reduce work-related stress, improve emotional well-being, and lower physiological stress indicators such as salivary cortisol levels [22]. Similarly, heat-treated (HT) forms of *Lacticaseibacillus paracasei* PS23 (PS23) have demonstrated psychobiotic potential, with both preclinical and clinical studies indicating reductions in anxiety- and depression-like symptoms [23,24] as well as lowered cortisol levels in highly stressed professionals such as nurses [25]. While both PS128 and HT-PS23 exhibit distinct psychobiotic properties, no clinical study has examined whether combining a live strain with a heat-treated strain may produce complementary or synergistic effects. This represents a key knowledge gap, particularly as these strains act through partially non-overlapping mechanisms, PS128 primarily through neuromodulatory pathways and HT-PS23 through immune and stress-related signaling. Firefighters represent a uniquely vulnerable group due to a combination of life-threatening responsibilities, irregular schedules, administrative demands, and frequent emergency deployments [26]. These cumulative and persistent stressors place firefighters at particularly high risk for chronic stress and associated psychological outcomes including anxiety, emotional exhaustion and reduced well-being. To address the identified gap, this study investigated whether the combined use of live PS128 and heat-treated PS23 offers additive or complementary benefits in alleviating psychological stress and emotional disturbances in firefighters. Using validated psychometric instruments and biochemical markers, we aimed to provide the first clinical evidence evaluating this dual-strain psychobiotic strategy in a high-stress professional population.

## 2. Materials and Methods

### 2.1. Study Design

An effect size of d = 0.29 was reported in a previous meta-analysis of healthy populations [27]. Based on this, a sample size of 96 was required to achieve 80% power with an α of 0.05. To account for an estimated 20% attrition rate, a total of 120 participants were recruited. All participants participated in this double-blind randomized controlled trial. Based on their registration codes on clinicaltrials.gov (Identifier: NCT06381479), subjects were randomly allocated to one of two groups: the psychobiotic group receiving Neuralli Mood (PS128 combined with HT-PS23) or the placebo group. The random allocation sequence was generated using a computer-based random number generator with a 1:1 allocation ratio. Block randomization with a fixed block size of four was employed to ensure balanced group sizes. Allocation concealment was maintained using sequentially numbered, opaque, sealed envelopes prepared by an independent researcher not involved in participant enrollment or assessment.

Participants were eligible for inclusion if (1) they were 20–60 years old; and (2) reported a perceived stress scale (PSS) score ≥ 14. Individuals were excluded if (1) they had used antibiotics or probiotic products (powder, capsule, or tablet forms) within the previous month; (2) were currently taking Chinese or Western medications for severe acute conditions; (3) had uncontrolled hypertension or diabetes, a history of cancer, or a diagnosed mental illness; (4) pregnancy or breastfeeding, participation in any other interventional clinical study within the past three months; or (5) any conflict of interest with the principal investigator (e.g., students under the PI’s supervision) or circumstances deemed unsuitable for participation, such as inability to read or understand the consent form.

The placebo capsules contained microcrystalline cellulose, while each psychobiotic capsules included powder composed of PS128 (1.5 × 10^10^ CFU) and HT-PS23 (1 × 10^10^ cells). Both groups consumed two capsules daily after dinner for a total of 8 weeks. To ensure blinding, the acid-resistant capsules for both groups were identical in appearance, including size, color, texture, and odor. Participants, outcome assessors, and data analysts were all blinded to group assignments. Study products were labeled and dispensed according to randomization codes by personnel not involved in outcome assessment or data analysis. The study products were provided by Bened Biomedical Co., Ltd. (Taipei, Taiwan) without compensation.

**Figure 1:**
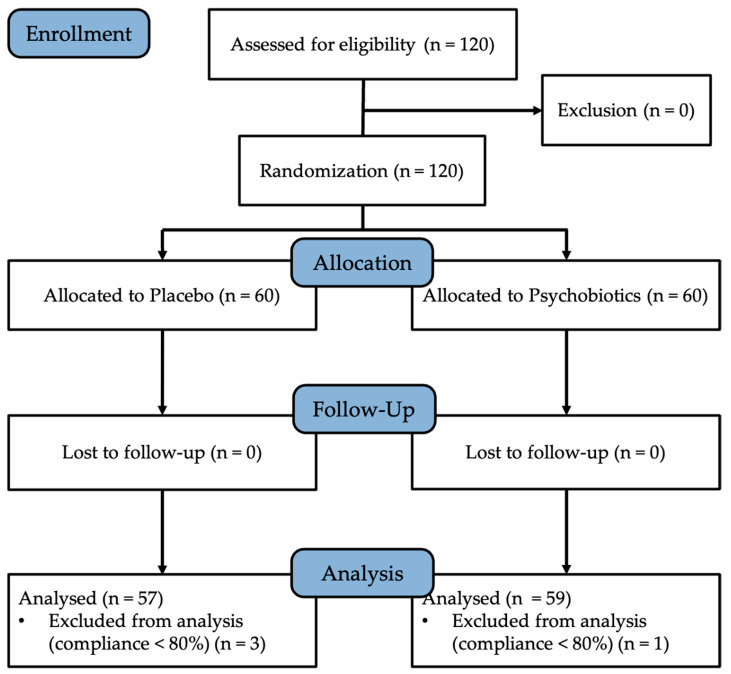
CONSORT diagram of the study.

All outcome measures were conducted before and after the 8-week supplementation period, including stress-related questionnaire assessments and analysis of blood biomarkers. Participants whose compliance with the assigned supplementation regimen was less than 80%, as calculated by the number of remaining capsules in returned bottles, were excluded from the final analyses. After exclusion, the data from 57 and 59 participants in the placebo and psychobiotic groups, respectively, were included in the outcome evaluation. The participant flow diagram, including the interventions and assessments, are shown in Figure 1.

### 2.2. The Perceived Stress Scale, PSS

The PSS is a widely used questionnaire to evaluate self-perceived stress responses over the past month [28,29]. The total PSS score was obtained by summing the scores for the 14 items. The scores range from 0 to 56, with a higher score indicating a higher perception of stress.

### 2.3. The Job Stress Scale, JSS

Work-related stress was measured using the Job Stress Scale (JSS), which was translated into traditional Chinese by the Ministry of Labor of Taiwan [30]. The JSS is a self-reported scale with 38 items on a 5-point Likert scale evaluating respondents’ job stress, control over job, burden, interpersonal relationships, satisfaction, psychological health, energy level and general health. The subscale scores are weighted according to the number of items in the range of 0 to 100, with higher scores indicating lower work-related stress.

### 2.4. The Chinese Version of the Copenhagen Burnout Inventory, C-CBI

The Copenhagen Burnout Inventory (CBI) was translated and modified into a Chinese version (C-CBI) by Yeh and colleagues, which mainly focused on personal (6 items) and work-related burnout (7 items) [31]. The respondents were asked to answer 13 questions on how frequently they experienced exhaustion on a 5-point Likert scale. The scores were transformed into a range of 0 to 100, with higher scores indicating greater levels of perceived overwork or burnout.

### 2.5. The State-Trait Anxiety Inventory, STAI

The State-Trait Anxiety Inventory (STAI) was administered to evaluate transient anxiety levels and persistent anxiety tendency [32]. The ‘state’ and ‘trait’ of the STAI are composed of 20 items, separately. Two factors (present and absent) in each subscale have been identified and confirmed in a Taiwanese population [33]. The total score ranged from 40 to 160. Higher scores indicate greater anxiety levels.

### 2.6. The Depression Anxiety and Stress Scale-42, DASS-42

The Depression Anxiety and Stress Scale-42 (DASS-42) is a 42-item questionnaire to measure depressive, anxious, and stressful symptoms over the past week on a 4-point Likert scale [34]. Each subscale score was calculated as the sum of the 14 items. The total score for each subscale ranges from 0 to 42. Higher scores indicated worse psychological symptoms.

### 2.7. The Insomnia Severity Index, ISI

The Insomnia Severity Index (ISI) is a well-developed instrument for measuring insomnia symptoms, and its Chinese version was translated by Yang et al. with good reliability and validity [35]. The total ISI score ranged from 0 to 28, with higher scores indicating more severe insomnia.

### 2.8. The Short Form of Quality of Life, Enjoyment, and Satisfaction Questionnaire, QLESQ-SF

The quality of life was assessed by the Short Form of Quality of Life, Enjoyment, and Satisfaction Questionnaire (QLESQ-SF) [36]. The QLESQ-SF is a 16-item questionnaire that asks respondents to rate their psychological and physical well-being on a 5-point Likert scale. The total score on the QLESQ-SF was the sum of the first 14 items, with scores ranging from 14 to 70. Higher scores indicate a better quality of life.

### 2.9. The Visual Analogue Scale of Gastrointestinal Discomfort, VAS-GI

The Visual Analogue Scale of Gastrointestinal Discomfort (VAS-GI) is a self-report questionnaire that assesses the severity of gastrointestinal discomfort [22]. Respondents were asked to rate symptoms, including dry mouth, swallowing, loss of appetite, nausea and vomiting, bloating, stomach ache, upper abdominal pain, lower abdominal pain, constipation, and diarrhea on a 10 cm line. Participants received brief training on how to mark symptom severity on the 10 cm visual analogue line, with a practice example provided to promote consistent use of the scale. Each symptom score ranges from 0 to 10, with an aggregate score ranging from 0 to 100. Higher scores indicated more severe symptoms.

### 2.10. The Sleep Diary

Sleep hygiene was evaluated by using a daily sleep diary. Participants were given standardized instructions on how to complete the sleep diary, including examples of sleep onset, wake time, and daily rating items, to ensure consistent reporting. Sleep onset time, wakeup time, nightmares percentage, enough sleep percentage, and snoozing percentage were recorded daily after waking up, with an overall sleep score ranging from 0 to 100. At least three consecutive days per week were required to calculate the average score.

### 2.11. The Patient Global Impression of Change, PGI-C

The Patient Global Impression of Change (PGI-C) is a self-reported inventory with items rated on a 7-point Likert scale [37]. The respondents were asked to evaluate their overall subjective changes following the intervention. The PGI-C score ranges from 1 to 7, with 4 indicating no changes. Lower scores indicated greater improvement; conversely, higher scores indicated greater deterioration.

### 2.12. Blood Biomarkers

Blood samples were collected from all participants at consistent time points, not in a fasted state, for each individual at baseline and after the 8-week intervention. All blood samples were collected between 8:00 and 10:00 a.m., after participants arrived at the fire station and before beginning their daily operational duties, to minimize circadian variability in cortisol and ACTH. Under standardized conditions, blood was drawn from the antecubital vein by qualified nursing personnel. The collected samples were processed by centrifugation at 1500× *g* for 15 min at 4 °C to isolate serum. The serum was subsequently aliquoted and stored at −80 °C until further analysis. Biochemical assays were performed to evaluate the neuroendocrine stress markers. Serum cortisol (07027150190, e801, Roche Diagnostics, Basel, Switzerland), adrenocorticotropic hormone (ACTH) (E-EL-H0137, Elabscience, Houston, TX, USA), and norepinephrine (E-EL-0047, Elabscience, Houston, TX, USA) concentrations were quantified using commercially available enzyme-linked immunosorbent assay (ELISA) kits according to the manufacturer’s protocols. These biomarkers were selected to reflect HPA axis activity and sympathetic nervous system response, providing objective measures to complement self-reported stress and emotional indices.

### 2.13. Statistical Analysis

Categorical variables were expressed by percentage, and continuous variables were expressed by mean ± SD. We used independent *t*-tests and Pearson’s chi-squared tests to examine demographic characteristics and baseline indicators. Repeated measures ANOVA and generalized estimating equations (GEE) were used to investigate efficacy. GEE was chosen because it provides population-averaged estimates, is robust to violations of sphericity and normality assumptions, and can accommodate unbalanced repeat-ed-measure data and missingness. Both sex and age were found to be important factors in occupational stress [38,39]. Therefore, sex and age were used as covariates in the GEE analyses. The Bonferroni method was used to adjust multiple comparisons. The standardized coefficient, 95% confidence interval (CI), and *p*-values were reported. Statistical analyses were performed using SPSS version 20.0. The significance level was set at *p* = 0.05.

## 3. Results

### 3.1. Demographic Characteristics and Baseline Performances

A summary of baseline characteristics is provided below to confirm group comparability before the intervention. A total of 120 participants were randomized into two groups: placebo (*n* = 60) and psychobiotics (*n* = 60). All participants completed the 8-week trial; however, based on capsule count for compliance assessment, 116 participants (placebo, *n* = 57; psychobiotics, *n* = 59) were included in the final analysis. No significant differences were observed in the baseline demographics or questionnaire scores between the groups, indicating comparability at study entry (Table 1 and Table S1). Additionally, no adverse events were reported during the study period.

### 3.2. Impact of Psychobiotic Intervention on Psychological Parameters

Key findings from each questionnaire are summarized below, followed by detailed item-level and scale-level results for clarity. As shown in Table 2, no significant group-by-time interaction was observed between the two groups on the PSS total score (*β* = −0.293, *p* = 0.694, 95% CI [−1.749, 1.164], Cohen’s d = 0.16). However, the psychobiotics group showed a significant improvement in the JSS general health compared with the placebo group (*β* = 1.848, *p* = 0.025, 95% CI [0.236, 3.460], Cohen’s d = 0.89). Moreover, the psychobiotics showed a significant reduction in anxious symptoms compared with the placebo, especially in the STAI state anxiety-absent subscale (*β* = −2.472, *p* = 0.006, 95% CI [−4.233, −0.712], Cohen’s d = 0.43). Table 2 shows a significant group-by-time interaction for the ISI total score (*β* = –1.609, *p* = 0.041, 95% CI [–3.153, –0.065], Cohen’s d = 0.46), indicating a greater reduction in insomnia symptoms in the psychobiotics group compared with the placebo group. Furthermore, the early awakening index in the ISI also showed a significant reduction in the psychobiotics group (*β* = −2.224, *p* = 0.048, 95% CI [−4.427, −0.022], Cohen’s d = 0.56). No significant interaction effects were observed for other indicators (Table S2). The PGI-C results showed no significant difference with *p* = 0.250, Cramer’s V = 0.21 (Table 3).

### 3.3. Effects of Psychobiotic Supplementation on Stress-Associated Blood Biomarkers

**Figure 2:**
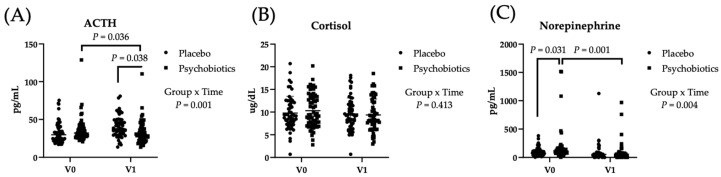
Effect of psychobiotic supplementation on (**A**) ACTH, (**B**) cortisol, and (**C**) norepinephrine. Data are shown as mean ± SD. Presented a significant effect by repeated measures ANOVA (*p* < 0.05). Placebo *n* = 57, Psychobiotics *n* = 59.

Overall, changes in stress-related biomarkers were consistent with improvements observed in questionnaire outcomes. Blood biomarker levels were compared between the baseline (V0) and the post-intervention (V1) across different groups (Figure 2). A significant group-by-time interaction was observed for adrenocorticotropic hormone (ACTH) levels over the 8-week administration period (*p* = 0.001). Further post hoc analysis indicated that ACTH levels in the psychobiotics group at V1 were significantly lower than those in the placebo group at V1 (*p* = 0.038) and the psychobiotics group at V0 (*p* = 0.036). However, the difference in cortisol levels between the psychobiotic and placebo groups was not statistically significant. Additionally, norepinephrine levels showed a significant group-by-time interaction (*p* = 0.004), and post hoc analysis confirmed that norepinephrine levels in the psychobiotics group at V1 were significantly lower than those at V0 (*p* = 0.001).

## 4. Discussion

This randomized, double-blind, placebo-controlled clinical trial demonstrated that an 8-week supplementation with PS128 and HT-PS23 produced significant group × time interactions for specific subscales of mental health assessments, including JSS general health, STAI state anxiety absent, ISI early awakening, and ISI total, while concurrently reducing ACTH and norepinephrine concentrations. The intervention was well tolerated, and no adverse events were reported by the participants throughout the study period. The integration of questionnaire-based assessments and physiological biomarkers offers converging evidence for the psychobiotic efficacy of this dual-strain intervention. These findings contribute to the growing body of literature suggesting that targeted probiotic or postbiotic supplementation can support mental well-being in populations experiencing sustained psychological load [2,40].

Cortisol is a central output of HPA axis activation, yet psychobiotic interventions show considerable variability in cortisol responses across human trials [16,27,41,42,43]. In the present study, ACTH and norepinephrine decreased while cortisol remained unchanged, an ACTH–cortisol dissociation that has been previously described in chronically stressed or dysregulated HPA states, where adrenal responsiveness to ACTH may be reduced [44,45,46]. Rather than emphasizing mechanistic complexity, these findings underscore the translational relevance of psychobiotics as a practical approach to supporting psychological resilience in high-stress occupations such as firefighting [26]. Interpretation of the underlying biological pathways, however, is limited by the absence of microbiota profiling, SCFA analyses, cytokine markers, and dietary control, as well as the predominance of male participants, factors that should be addressed in future mechanistic or mixed-sex cohort studies [38,39,47].

Beyond the modulation in the endocrine axis, the emotional improvements observed in our study, particularly in group × time interactions of JSS total and ISI total as well as STAI state anxiety absent, may involve microbial influences on neurotransmitter availability and signaling. In preclinical models, PS128 was shown to elevate central serotonin and dopamine levels in rodents, suggesting that PS128 can directly modulate neurotransmitter pathways involved in mood and stress regulation [19]. Although the neurotransmitter levels were not measured in this study, the concurrent reduction in overall job stress perception, state anxiety, and insomnia severity may support the hypothesis of enhanced monoaminergic activity and, possibly, GABAergic tone, as suggested by previous reports of probiotic modulation of GABA pathways [48]. In addition to HPA-axis and sympathetic modulation, psychobiotics may also influence psychological outcomes through vagal and neurotransmitter-related pathways that operate independently of cortisol. Several *Lactobacillus* and *Bifidobacterium* strains have been shown to activate vagal afferents, thereby altering limbic circuit activity and stress-related behaviors [48]. Microbial metabolites and structural components can modulate monoaminergic signaling by promoting serotonin, dopamine, or GABA availability through interactions with enterochromaffin cells or by altering tryptophan metabolic flux [19,48,49]. SCFAs and other microbial metabolites may further support GABAergic tone and neuroimmune balance [47,50]. These neuromodulatory effects may provide an alternative explanation for the reductions in anxiety, job stress, and insomnia observed in our study, even when cortisol remained unchanged. Given that PS128 exerts dopaminergic and serotonergic activity [19,20,21], and HT-PS23 modulates immune–neural signaling through pathways such as TLR2-IL-10-TNF-α and mucosal immune regulation [23,24,51,52,53,54,55], their combined actions may converge on multiple neural circuits beyond classical HPA-axis regulation.

In addition to PS128, an important component of the psychobiotic formulation used in this study was HT-PS23 [23], which remained biologically active possibly due to its microbe-associated molecular patterns [51]. These components, including peptidoglycans and lipoteichoic acids, interact with Toll-like receptors (especially TLR2) in intestinal epithelial and immune cells [52,53]. Activation of these pathways can induce regulatory immune responses, notably IL-10 release and suppression of pro-inflammatory cytokines such as TNF-α and IL-6 [54]. Chronic psychological stress has been linked to inflammatory mediators, which can further activate the HPA axis. This activation, along with the action of inflammatory mediators, can impair neurotransmitter balance, potentially through mechanisms such as the kynurenine pathway [49]. By modulating gut-associated immunity and maintaining mucosal barrier integrity, HT-PS23 likely contributes to the reduction in systemic inflammation and the restoration of immune–neuroendocrine homeostasis [55].

Insomnia is frequently precipitated by stressful life events, as stress-induced physiological and cognitive hyperarousal can disrupt sleep processes and contribute to the development of chronic insomnia [56]. In line with previous findings showing that PS128 improves sleep in individuals with self-reported insomnia [21], our study also observed significant sleep-related improvements, as indicated by reduced ISI scores. Insomnia both contributes to and results from stress-related disorders, influenced by glucocorticoid dysregulation, inflammatory signaling, and neurotransmitter imbalances [57]. SCFAs such as butyrate, produced by microbial fermentation of dietary fiber, have been shown to cross the blood–brain barrier, enhance GABAergic signaling, and promote slow-wave sleep [47]. Although SCFA levels were not measured in this study, probiotic intervention may have modulated microbial communities toward greater butyrate production. This modulation could have mimicked the effects of SCFAs via immune and neural routes [50]. These microbiota-mediated mechanisms may underlie the sleep-related improvements observed in our trial and have been reflected in prior human studies [50]. The observed improvement in insomnia in this study may primarily be ascribed to PS128, given that the HT-PS23 intervention did not yield significant changes in sleep-related measures [25]. Taken together, these studies validate the utility of multi-strain psychobiotics and highlight that combining strains with overlapping or complementary mechanisms, whether live or heat-treated, may produce broader and more sustained clinical effects.

While our findings are largely consistent with existing psychobiotic literature, some discrepancies across studies warrant closer examination to better understand the context-dependent nature of probiotic efficacy. Notably, certain trials involving *Lacticaseibacillus casei* or *B. breve* strains have reported improvements in mood or sleep without accompanying changes in cortisol or systemic inflammatory markers [58,59,60]. These results highlight the challenge of attributing psychobiotic effects solely to the HPA axis suppression or cytokine regulation. In a study involving *L. casei* Shirota administered to patients with chronic fatigue syndrome, both mood and sleep quality improved significantly, despite no measurable reduction in serum cortisol levels, suggesting the possibility of peripheral immune modulation or vagal activation as alternative pathways of action [58]. In contrast, our trial demonstrated parallel improvements in subjective psychological indicators and ACTH reduction, pointing toward a more direct engagement in HPA axis regulation. The relatively homogeneous profile of our participants, characterized by elevated baseline perceived stress (PSS ≥ 14), may have further enhanced the detectability of psychobiotic effects compared to studies involving more heterogeneous or subclinical populations.

In summary, this study contributes to a growing body of evidence supporting the efficacy of targeted psychobiotic intervention in modulating stress-related outcomes. The combined use of PS128 with HT-PS23 yielded significant group x time interactions in mental health subscales, accompanied by reductions in ACTH and norepinephrine. These findings indicate a potential benefit spanning both psychological and physiological stress responses. While most psychobiotic trials to date have focused on live strains alone, our findings add to the limited but emerging evidence suggesting that the co-administration of live and heat-treated strains may offer synergistic or complementary benefits. Theoretical considerations and prior studies have proposed that co-administration of live and heat-treated probiotic strains may reduce microbial growth competition that can arise in live-only formulations [61,62].

The observed clinical outcomes in this study reinforce the validity of this formulation strategy and highlight its translational potential for stress management. Nonetheless, this study has several limitations. First, the study population consisted exclusively of firefighters, a decision made to reduce heterogeneity in occupational stress profiles, as different professions are known to present distinct psychosocial stressors. Although this design choice enhanced internal validity, it may limit the generalizability of our findings to broader populations. Second, the sample exhibited a notable sex imbalance due to the male-dominant composition of the firefighting profession, which may have influenced sex-specific responses to the intervention. Third, our assessments were limited to psychological questionnaires and serum biomarkers, without including microbial composition, gut-derived metabolites, or cytokine markers, which could further elucidate the underlying mechanisms. In addition, blood samples were collected in a non-fasted state, which may introduce variability in circulating hormone levels and should be considered a methodological limitation. Although all samples were drawn at approximately the same time of day to mitigate diurnal effects, future studies should incorporate standardized fasting conditions to reduce potential metabolic confounding. Future studies should expand upon these results by incorporating microbiota profiling, SCFA quantification, and inflammatory cytokine measurements to better characterize the strain-specific mechanisms of action. Despite these limitations, the present findings provide compelling evidence for the psychobiotic potential of PS128 and HT-PS23 in managing chronic stress and enhancing mental well-being.

## 5. Conclusions

This randomized, placebo-controlled trial provides clinical evidence that combined supplementation with PS128 and HT-PS23 can significantly improve psychological outcomes related to stress, including overall job stress perception, state anxiety and insomnia severity. These improvements were accompanied by significant reductions in ACTH and norepinephrine levels, suggesting that the intervention modulated both the HPA axis and sympathetic nervous system activities. Although psychobiotic studies have predominantly focused on live strains, our findings support the potential synergistic effects of combining live and heat-treated strains in stress management. Overall, this dual-strain psychobiotic formulation is a promising, safe, and well-tolerated strategy for improving stress-related mental health in high-risk populations.

## Figures and Tables

**Table 1:** Comparisons of baseline characteristics between the two groups.

	Placebo (*n* = 57)		Psychobiotics (*n* = 59)		
	*n*	%	*n*	%	*p*-Value
Sex					0.077
Male	54	95.0	50	85.0	
Female	3	5.0	9	15.0	
Marital status					0.265
Single	22	39.3	24	41.4	
Married	31	55.4	25	43.1	
Cohabit	3	5.4	8	13.8	
Divorced	0	0.0	1	1.7	
Children					0.198
With	29	50.9	23	39.0	
Without	28	49.1	36	61.0	
Occupation Experience					0.971
<1 year	3	5.3	4	6.8	
1~2 years	2	3.5	2	3.4	
2~4 years	2	3.5	5	8.5	
4~6 years	9	15.8	8	13.6	
6~8 years	4	7.0	5	8.5	
8~10 years	7	12.3	8	13.6	
10~15 years	9	15.8	8	13.6	
>15 years	21	36.8	19	32.2	
Other Supplement					0.060
With	6	10.5	14	23.7	
Without	51	89.5	45	76.3	
	Mean	SD	Mean	SD	*p*-value
Age	34.3	6.9	34.9	7.0	0.645
Years of Education	16.0	1.9	15.6	1.9	0.288
BMI	26.2	3.3	25.9	3.5	0.678

Continuous variables were analyzed by independent *t* tests, Categorial variables were analyzed by Pearson Chi-Squared tests.

**Table 2:** Effect of psychobiotic supplementation on psychological outcomes.

Variables	Group Effect		Time Effect		Group × Time	
	B (95% CI)	*p*-Value	B (95% CI)	*p*-Value	B (95% CI)	*p*-Value
Perceived Stress Scale (PSS)						
PSS Total	0.917 (−0.764, 2.597)	0.285	1.034 (0.206, 1.861)	0.014 *	−0.293 (−1.749, 1.164)	0.694
Job Stress Scale (JSS)						
Job Stress	0.000 (0.000, 0.000)	1.000	0.750 (−0.648, 2.149)	0.293	1.035 (−0.702, 2.772)	0.243
Control Over Job	0.301 (−1.736, 2.338)	0.772	−0.362 (−2.083, 1.359)	0.680	1.017 (−1.202, 3.236)	0.369
Job Burden	−0.660 (−2.707, 1.387)	0.527	−1.370 (−3.013, 0.274)	0.103	1.150 (−0.881, 3.182)	0.267
Interpersonal Relationships	0.862 (−1.275, 2.998)	0.429	−0.637 (−2.341, 1.067)	0.464	0.235 (−1.945, 2.415)	0.833
Job Satisfaction	1.477 (−0.363, 3.318)	0.116	1.021 (−1.047, 3.090)	0.333	−0.783 (−3.277, 1.712)	0.539
Psychological Health	0.464 (−1.472, 2.400)	0.639	0.308 (−0.881, 1.496)	0.612	0.187 (−1.459, 1.833)	0.824
Energy Level	0.934 (−0.869, 2.737)	0.310	−0.837 (−1.724, 0.051)	0.065	0.560 (−0.919, 2.040)	0.458
General Health	−0.116 (−2.172, 1.939)	0.912	−1.338 (−2.525, −0.150)	0.027 *	1.848 (0.236, 3.460)	0.025 *
Chinese version of Copenhagen Burnout Inventory (C-CBI)						
C-CBI Personal Burnout	0.876 (−1.142, 2.893)	0.395	−0.271 (−1.635, 1.093)	0.697	−0.040 (−1.868, 1.787)	0.966
C-CBI Work-Related Burnout	0.733 (−1.445, 2.910)	0.510	−0.046 (−1.545, 1.454)	0.952	0.068 (−1.920, 2.057)	0.946
State and Trait Anxiety Inventory (STAI)						
STAI State Anxiety Present	−0.206 (−2.105, 1.692)	0.831	1.059 (−0.927, 3.045)	0.296	−1.260 (−3.661, 1.141)	0.304
STAI State Anxiety Absent	0.311 (−1.326, 1.949)	0.709	2.091 (0.862, 3.321)	0.001 **	−2.472 (−4.233, −0.712)	0.006 **
STAI State	0.038 (−1.704, 1.780)	0.966	1.613 (−0.027, 3.254)	0.054	−1.912 (−4.001, 0.177)	0.073
STAI Trait Anxiety Present	−0.771 (−2.494, 0.953)	0.381	0.336 (−0.890, 1.563)	0.591	−0.289 (−1.958, 1.379)	0.734
STAI Trait Anxiety Absent	−0.117 (−2.113, 1.878)	0.908	1.448 (0.490, 2.407)	0.003 **	−1.101 (−2.415, 0.213)	0.100
STAI Trait	−0.512 (−2.282, 1.258)	0.571	0.865 (−0.074, 1.804)	0.071	−0.678 (−2.006, 0.651)	0.317
STAI Total	−0.243 (−1.972, 1.487)	0.783	1.327 (0.173, 2.480)	0.024 *	−1.393 (−3.015, 0.229)	0.092
Depression Anxiety and Stress Scale-42 (DASS-42)						
DASS-42 Depression	1.260 (−0.402, 2.922)	0.137	0.199 (−0.731, 1.129)	0.674	−0.120 (−1.460, 1.220)	0.861
DASS-42 Anxiety	0.752 (−1.134, 2.638)	0.435	0.841 (−0.375, 2.056)	0.175	−1.126 (−2.647, 0.395)	0.147
DASS-42 Stress	0.187 (−1.536, 1.910)	0.832	0.885 (−0.054, 1.823)	0.065	−1.056 (−2.452, 0.340)	0.138
Insomnia Severity Index (ISI)						
ISI Initiation	−0.347 (−2.211, 1.518)	0.716	0.286 (−1.626, 2.198)	0.770	−0.358 (−2.725, 2.010)	0.767
ISI Maintenance	−0.228 (−1.888, 1.432)	0.788	1.490 (−0.003, 2.983)	0.050 *	−1.078 (−3.005, 0.850)	0.273
ISI Early Awakening	−0.064 (−2.060, 1.933)	0.950	1.983 (0.177, 3.788)	0.031 *	−2.224 (−4.427, −0.022)	0.048 *
ISI Total	−0.309 (−2.069, 1.451)	0.731	2.195 (1.060, 3.329)	0.000 ***	−1.609 (−3.153, −0.065)	0.041 *
Short Form of Quality of Life, Enjoyment, and Satisfaction Questionnaire (QLESQ-SF)						
QLESQ-SF Overall	0.031 (−1.931, 1.993)	0.975	−1.443 (−2.920, 0.034)	0.056	2.081 (−0.149, 4.310)	0.067
QLESQ-SF Psychological	0.511 (−1.371, 2.393)	0.595	−0.911 (−2.126, 0.304)	0.142	1.797 (−0.259, 3.852)	0.087
QLESQ-SF Physical	0.336 (−1.478, 2.149)	0.717	−1.389 (−2.526, −0.253)	0.017 *	1.460 (−0.144, 3.064)	0.074
QLESQ-SF Total	0.499 (−1.382, 2.380)	0.603	−1.164 (−2.275, −0.054)	0.040 *	1.851 (−0.022, 3.725)	0.053

Continuous variables were analyzed by generalized estimating equation with age and sex as covariables. Results were presented in * *p* < 0.05, ** *p* < 0.01, *** *p* < 0.001.

**Table 3:** Effect of psychobiotics supplementation on PGI-C.

	Placebo		Psychobiotics		*p*-Value
	*n*	%	*n*	%	
Very much improved	1	1.8	0	0.0	0.250
Much improved	3	5.3	5	8.5	
Minimally improved	34	59.6	29	49.2	
No change	17	29.8	25	42.4	
Minimally worse	2	3.5	0	0.0	
Much worse	0	0.0	0	0.0	
Very much worse	0	0.0	0	0.0	

PGI-C was analyzed by Pearson Chi-Squared tests.

## Data Availability

The original contributions presented in this study are included in the article/Supplementary Materials. Further inquiries can be directed to the corresponding author.

## Supplementary Materials

The following supporting information can be downloaded at: https://www.mdpi.com/article/10.3390/foods14244190/s1. Table S1. Comparisons of questionnaire scoresb etween the two groups at baseline. Table S2. Effect of psychobiotic supplementation on gastrointestinal and sleep outcomes.
