# Supplementary material for: Dual-Strain Psychobiotics Combining Live Lactiplantibacillus plantarum PS128 and Heat-Treated Lacticaseibacillus paracasei PS23 Improve Psychological and Neuroendocrine Outcomes in Stressed Adults: A Randomized, Placebo-Controlled Trial
Source: Foods. 2025 Dec 6;14(24):4190. doi: 10.3390/foods14244190 (PMC12731655; doi:10.3390/foods14244190)
Supplement: Supplementary file 1 [file foods-14-04190-s001.zip › Supplementary Table S1.pdf]

Supplementary Table S1. Comparisons of questionnaire scores between the two groups at baseline

	Placebo (n = 57)		Psychobiotics (n = 59)		p-value
	n	%	n	%	
<u>Perceived Stress Scale (PSS)</u>					
PSS-14 Total	28.2	8.6	27.8	6.5	0.859
<u>Job Stress Scale (JSS)</u>					
Job Stress	63.2	30.9	63.4	32.8	1.000
Control Over Job	57.6	8.0	59.9	8.8	0.160
Job Burden	76.9	8.4	75.9	9.6	0.673
Interpersonal Relationships	71.7	7.9	75.3	10.2	0.132
Job Satisfaction	66.3	16.1	70.5	15.0	0.247
Psychological Health	54.0	15.3	54.9	15.3	0.831
Energy Level	42.3	17.9	44.5	16.5	0.397
General Health	36.1	8.0	35.1	8.8	0.589
<u>Chinese version of Copenhagen Burnout Inventory (C-CBI)</u>					
C-CBI Personal Burnout	48.8	18.6	49.9	16.4	0.849
C-CBI Work-Related Burnout	47.7	14.1	48.2	14.3	0.891
<u>State and Trait Anxiety Inventory (STAI)</u>					
STAI State Anxiety Present	21.5	5.6	21.7	6.0	0.518
STAI State Anxiety Absent	22.8	5.4	22.3	4.8	0.944
STAI State	44.2	10.6	44.1	10.3	0.746
STAI Trait Anxiety Present	28.2	5.0	28.1	5.6	0.987
STAI Trait Anxiety Absent	22.2	4.6	22.0	4.0	0.934
STAI Trait	50.4	9.1	50.1	9.1	0.977
STAI Total	94.7	19.0	94.2	18.1	0.890
<u>Depression Anxiety and Stress Scale-42 (DASS-42)</u>					
DASS-42 Depression	11.1	6.9	8.9	6.9	0.156
DASS-42 Anxiety	10.2	5.8	9.1	6.3	0.452
DASS-42 Stress	17.4	8.1	17.4	8.5	0.907
<u>Insomnia Severity Index (ISI)</u>					
ISI Initiation	1.4	1.0	1.3	0.9	0.508
ISI Maintenance	2.0	1.2	1.8	1.1	0.633

	Placebo ($n = 57$)		Psychobiotics ($n = 59$)		p-value
	n	%	n	%	
ISI Early Awakening	1.5	1.1	1.4	1.1	0.936
ISI Total	12.8	5.4	12.3	5.2	0.627
Short Form of Quality of Life, Enjoyment, and Satisfaction Questionnaire (QLESQ-SF)					
QLESQ-SF Overall	3.3	0.7	3.3	0.6	0.502
QLESQ-SF Psychological	33.0	5.5	32.6	5.0	0.395
QLESQ-SF Physical	14.7	2.3	14.5	2.6	0.651
QLESQ-SF Total	47.7	7.1	47.2	6.9	0.426
Visual Analog Scale for Gastrointestinal discomfort (VAS-GI)					
VAS-GI Dry Mouth	3.5	2.8	3.1	2.5	0.636
VAS-GI Swallowing	0.9	1.6	0.8	1.7	0.911
VAS-GI Losing Appetite	1.9	2.2	1.5	1.8	0.345
VAS-GI Nausea & Vomiting	0.8	1.4	0.7	1.5	0.847
VAS-GI Bloating	2.5	2.4	2.4	2.9	0.973
VAS-GI Stomachache	2.3	2.7	1.8	2.6	0.447
VAS-GI Upper Abdominal Pain	1.0	1.6	0.9	1.7	0.736
VAS-GI Lower Abdominal Pain	1.0	1.7	0.9	1.6	0.911
VAS-GI Constipation	0.9	1.8	1.2	2.2	0.666
VAS-GI Diarrhea	1.9	2.6	1.9	2.4	0.973
VAS-GI Total	16.8	14.4	15.2	13.5	0.694
Sleep Diary					
Sleep Diary Sleep Duration	428.2	66.9	434.2	61.3	0.620
Sleep Diary Sleep Duration SD	75.2	59.2	76.2	41.0	0.916
Sleep Diary Overall	71.1	11.9	72.3	10.7	0.549
Sleep Diary Nightmare (%)	25%	32%	12%	25%	0.083
Sleep Diary Enough Sleep (%)	29%	34%	38%	32%	0.268
Sleep Diary No Snooze (%)	34%	35%	31%	32%	0.235

Continuous variables were analyzed by independent t tests, Categorial variables were analyzed by Pearson Chi-Squared tests.
