# Supplementary material for: Dual-Strain Psychobiotics Combining Live Lactiplantibacillus plantarum PS128 and Heat-Treated Lacticaseibacillus paracasei PS23 Improve Psychological and Neuroendocrine Outcomes in Stressed Adults: A Randomized, Placebo-Controlled Trial
Source: Foods. 2025 Dec 6;14(24):4190. doi: 10.3390/foods14244190 (PMC12731655; doi:10.3390/foods14244190)
Supplement: Supplementary file 1 [file foods-14-04190-s001.zip › Supplementary Table S2.pdf]

Supplementary Table S2. Effect of psychobiotic supplementation on gastrointestinal and sleep outcomes

Variables	Group effect		Time effect		Group*Time	
	B (95%CI)	*p*-value	B (95%CI)	*p*-value	B (95%CI)	*p*-value
<u>Visual Analogue Scale for Gastrointestinal Discomfort (VAS-GI)</u>						
VAS-GI Dry Mouth	0.870 (-0.957, 2.697)	0.350	0.133 (-1.454, 1.720)	0.870	-0.193 (-2.109, 1.723)	0.844
VAS-GI Swallowing	0.427 (-1.051, 1.905)	0.571	0.209 (-0.491, 0.909)	0.558	0.217 (-1.253, 1.687)	0.772
VAS-GI Losing Appetite	-1.036 (-2.908, 0.836)	0.278	0.298 (-1.501, 2.097)	0.745	1.271 (-1.250, 3.793)	0.323
VAS-GI Nausea & Vomiting	-0.307 (-2.324, 1.711)	0.766	0.106 (-0.719, 0.932)	0.801	0.313 (-1.128, 1.754)	0.670
VAS-GI Bloating	-0.550 (-2.495, 1.394)	0.579	0.911 (-0.158, 1.979)	0.095	-0.889 (-2.355, 0.577)	0.235
VAS-GI Stomachache	1.113 (-0.402, 2.628)	0.150	0.685 (-0.122, 1.492)	0.096	-0.276 (-1.787, 1.234)	0.720
VAS-GI Upper Abdominal Pain	0.167 (-1.947, 2.282)	0.877	2.028 (0.590, 3.466)	0.006**	-1.360 (-3.740, 1.020)	0.263
VAS-GI Lower Abdominal Pain	-0.388 (-1.515, 0.739)	0.500	1.206 (0.187, 2.224)	0.020*	-0.353 (-2.429, 1.724)	0.739
VAS-GI Constipation	0.147 (-0.889, 1.184)	0.781	-0.300 (-1.363, 0.763)	0.580	-0.013 (-1.651, 1.625)	0.987
VAS-GI Diarrhea	1.556 (-0.225, 3.338)	0.087	1.393 (0.001, 2.784)	0.050*	-1.530 (-3.400, 0.341)	0.109
VAS-GI Total	0.458 (-1.247, 2.162)	0.599	1.011 (0.087, 1.934)	0.032*	-0.516 (-2.100, 1.068)	0.523
<u>Sleep Diary</u>						
Sleep Diary Sleep Duration	0.058 (-1.774, 1.891)	0.950	-1.617 (-3.660, 0.426)	0.121	1.838 (-0.444, 4.120)	0.114
Sleep Diary Sleep Duration SD	0.465 (-1.521, 2.452)	0.646	-2.416 (-4.572, -0.26)	0.028*	1.384 (-1.139, 3.907)	0.282
Sleep Diary Nightmare	-0.240 (-2.100, 1.621)	0.801	-0.230 (-1.692, 1.231)	0.757	0.117 (-1.547, 1.781)	0.890
Sleep Diary Enough Sleep	-0.060 (-2.153, 2.033)	0.955	-0.305 (-2.466, 1.855)	0.782	0.099 (-2.308, 2.507)	0.936
Sleep Diary No Snooze	0.821 (-1.187, 2.829)	0.423	0.333 (-1.333, 1.999)	0.695	-0.556 (-2.678, 1.566)	0.608
Sleep Diary Overall	-0.897 (-2.561, 0.768)	0.291	0.500 (-1.600, 2.600)	0.641	-0.544 (-2.785, 1.697)	0.634

Continuous variables were analyzed by generalized estimating equation with age and sex as covariables. Results were presented in $^{*}p < 0.05$, $^{**}p < 0.01$.
